# Supplementary material for: Spontaneously Reported Adverse Drug Reactions and Their Description in Hospital Discharge Reports: A Retrospective Study
Source: J Clin Med. 2021 Jul 26;10(15):3293. doi: 10.3390/jcm10153293 (PMC8348023; doi:10.3390/jcm10153293)
Supplement: Supplementary file 1 [file jcm-10-03293-s001.zip › jcm-1296883-supplementary.pdf]

Supplementary material

Table S1. Adverse drug reactions and involved drugs.

Adverse drug reactions (363)	Drugs involved (627; 209 different drugs)
**Nervous system disorders (63). Drugs involved (116) (62 different drugs)**	
Somnolence (8). Drugs involved (14)	quetiapine (2), lorazepam (2), paracetamol, ibuprofen, clonazepam, lithium, lormetazepam, phenobarbital, diazepam, risperidone, chloral
Depressed level of consciousness (6). Drugs involved (16)	quetiapine (4), lorazepam, diazepam, clonazepam, mirtazapine, lithium, haloperidol, fluoxetine, topiramate, methadone, clomethiazole, carbocisteine,
Coma (4). Drugs involved (10)	quetiapine (2), gabapentin, diazepam, lorazepam, lormetazepam, paliperidone, paroxetine, risperidone, tramadol
Encephalopathy (4). Drugs involved (7)	ertapenem (3), quetiapine, valproic acid, tacrolimus, meropenem
Ataxia (3). Drugs involved (3)	lorazepam, chloral hydrate, bupropion
Cerebral hemorrhage (3). Drugs involved (3)	acenocoumarol (2), rivaroxaban
Tremor (3). Drugs involved (6)	clonazepam, lithium, lormetazepam, phenobarbital, trazodone, bupropion
Guillain-Barre syndrome (2). Drugs involved (4)	measles, combinations with mumps and rubella, live attenuated; hepatitis A, inactivated, whole virus; raltitrexed, oxaliplatin
Neuropathy peripheral (2). Drugs involved (2)	bortezomib, benznidazole
Seizure (2). Drugs involved (2)	fluoxetine, busulfan
Serotonin syndrome (2). Drugs involved (6)	mirtazapine (2), aripiprazole, quetiapine, sertraline, venlafaxine
Anticholinergic syndrome (1). Drugs involved (1)	glycopyrronium bromide
Aphasia (1). Drugs involved (2)	propofol, sevoflurane
Cerebral hematoma (1). Drugs involved (2)	acetylsalicylic acid, edoxaban
Disorientation (1). Drugs involved (4)	dexketoprofen, diclofenac, ibuprofen, naproxen
Dyskinesia (1). Drugs involved (3)	haloperidol, quetiapine, trazodone
Dystonia (1). Drugs involved (2)	propofol, sevoflurane
Headache (1). Drugs involved (2)	omeprazole, fosfomycin
Horner's syndrome (1). Drugs involved (1)	bupivacaine
Hyperreflexia (1). Drugs involved (4)	clonazepam, phenobarbital, lithium, lormetazepam
Idiopathic intracranial hypertension (1). Drugs involved (1)	tretinoin
Leukoencephalopathy (1). Drugs involved (1)	methotrexate
Loss of consciousness (1). Drugs involved (1)	antithymocyte immunoglobulin
Meningitis aseptic (1). Drugs involved (2)	metamizole sodium, dexketoprofen
Mixed incontinence, urge and stress (1). Drugs involved (2)	quetiapine, risperidone
Myasthenia gravis (1). Drugs involved (1)	alemtuzumab
Myoclonus (1). Drugs involved (3)	clonazepam, haloperidol, quetiapine
Paraesthesia (1). Drugs involved (2)	omeprazole, fosfomycin

Progressive multifocal leukoencephalopathy (1). Drugs involved (1)	natalizumab
Status epilepticus (1). Drugs involved (1)	ertapenem
Subdural hematoma (1). Drugs involved (2)	acenocoumarol, levofloxacin
Syncope (1). Drugs involved (1)	tramadol
Thrombotic cerebral infarction (1). Drugs involved (1)	norethisterone
Transient ischemic attacks (1). Drugs involved (1)	danazol
Hypotonia (1). Drugs involved (2)	propofol, sevoflurane
**Metabolism and nutrition disorders (38). Drugs involved (72) (30 different drugs)**	
Lactic acidosis (11). Drugs involved (13)	metformin (9), furosemide, ibuprofen, nitroprusside, salbutamol
Metabolic acidosis (6). Drugs involved (16)	sulfamethoxazole and trimethoprim (5), tacrolimus (3), azithromycin (2), furosemide, methylprednisolone, piperacil·lina-tazobactam, ciclosporin, losartan, spironolactone
Hyperkalemia (6). Drugs involved (17)	sulfamethoxazole and trimethoprim (3), ibuprofen, dexketoprofen, tacrolimus, furosemide, azithromycin, enalapril, piperacillin and beta-lactamase inhibitor, ciclosporin, naproxen, losartan, diclofenac, propranolol, flecainide, spironolactone
Hypomagnesaemia (3). Drugs involved (4)	omeprazole (3), metformin
Inappropriate antidiuretic hormone secretion (3). Drugs involved (8)	quetiapine (2), lorazepam, haloperidol, carbamazepine, losartan and diuretics, zolpidem, citalopram
Hyperlactacidemia (2). Drugs involved (2)	salbutamol (2)
Hyponatremia (2). Drugs involved (3)	omeprazole, losartan and diuretics, linezolid
Hypercalcemia (2). Drugs involved (3)	calcifediol, calcitriol, calcium acetate
Hypervitaminosis D (1). Drugs involved (1)	calcifediol
Mitochondrial toxicity (1). Drugs involved (2)	propofol, sevoflurane
Hypoglycemia (1). Drugs involved (3)	methotrexate, pegaspargase, mercaptopurine
**Gastrointestinal disorders (36). Drugs involved (52) (38 different drugs)**	
Vomiting (9). Drugs involved (15)	paracetamol (3), ibuprofen, amiodarone, clonazepam, azithromycin, lithium, lormetazepam, phenobarbital, diazepam, carbocisteine, brivaracetam, amoxicillin, gadobutrol
Diarrhea (5). Drugs involved (5)	amiodarone, mycophenolic acid, nivolumab, everolimus, duloxetine
Retroperitoneal hematoma (3). Drugs involved (6)	acenocoumarol (2), omeprazole, rivaroxaban, allopurinol, sertraline
Pancreatitis (3). Drugs involved (3)	pegaspargase (2), apixaban
Aphthous ulcer (2). Drugs involved (4)	paracetamol, ibuprofen, amoxicillin and beta-lactamase inhibitor, sorafenib
Dyspepsia (2). Drugs involved (2)	azithromycin (2)
Abdominal pain (1). Drugs involved (1)	mycophenolic acid
Abdominal pain upper (1). Drugs involved (1)	paracetamol

Enterocolitis hemorrhagic (1). Drugs involved (1)	dasatinib
Gastrointestinal inflammation (1). Drugs involved (1)	methotrexate
Ileus paralytic (1). Drugs involved (1)	bortezomib
Nausea (1). Drugs involved (1)	tramadol
Neutropenic colitis (1). Drugs involved (2)	cyclophosphamide, docetaxel
Pancreatitis necrotising (1). Drugs involved (3)	methotrexate, pegaspargase, mercaptopurine
Peptic ulcer perforation (1). Drugs involved (3)	dexketoprofen, diclofenac, dexamethasone
Upper gastrointestinal hemorrhage (1). Drugs involved (1)	cabozantinib
Ulcerative gastritis (1). Drugs involved (1)	nivolumab
Sprue-like enteropathy (1). Drugs involved (1)	olmesartan medoxomil
**Hepatobiliary disorders (35). Drugs involved (69) (50 different drugs)**	
Hepatic cytolysis (17). Drugs involved (33)	amoxicillin and beta-lactamase inhibitor (4), atorvastatin (2), meropenem (2), paracetamol (2), ticagrelor, methylprednisolone, ceftazidime, sulfamethoxazole and trimethoprim, ibuprofen,
Hepatitis fulminant (7). Drugs involved (13)	paracetamol (3), isoniazid (2), omeprazole, metamizole sodium, dexketoprofen, enoxaparin, nivolumab, pyrazinamide, ipilimumab, repaglinide
Hepatitis cholestatic (6). Drugs involved (15)	atorvastatin (2), amoxicillin and beta-lactamase inhibitor, azathioprine, canagliflozin, clopidogrel, cloxacillin, dapagliflozin, enalapril, fluoxetine, metformin, mirtazapine, nifedipine, ranitidine,
Mixed liver injury (3). Drugs involved (5)	ceftazidime, ciprofloxacin, losartan, ticagrelor, tobramycin
Autoimmune hepatitis (1). Drugs involved (2)	interferon beta-1a, methylprednisolone
Hypertransaminasemia (1). Drugs involved (1)	labetalol
**Blood and lymphatic system disorder (31). Drugs involved (59) (45 different drugs)**	
Pancytopenia (6). Drugs involved (19)	metamizole sodium (3), methotrexate (3), teicoplanin, sulfamethoxazole and trimethoprim,
Agranulocytosis (5). Drugs involved (5)	ampicillin, docetaxel, metamizole sodium, paclitaxel, valganciclovir
Eosinophilia (4). Drugs involved (7)	clindamycin (2), quetiapine, ertapenem, piperacillin and beta-lactamase inhibitor, ceftriaxone, allopurinol
Neutropenia (3). Drugs involved (5)	cefotaxime, dexamethasone, metamizole sodium, quetiapine, vancomycin
Hemophagocytic lymphohistiocytosis (2). Drugs involved (2)	alemtuzumab, etanercept
Anemia megaloblastic (1). Drugs involved (5)	sulfamethoxazole and trimethoprim, omeprazole, gabapentin, carbamazepine, pregabalin
Aplasia pure red cell (1). Drugs involved (1)	azathioprine
Autoimmune hemolytic anemia (1). Drugs involved (2)	sulfamethoxazole and trimethoprim, tacrolimus
Bicytopenia (1). Drugs involved (1)	ceftaroline fosamil
Lymphopenia (1). Drugs involved (1)	alemtuzumab
Thrombocytopenic purpura (1). Drugs involved (2)	valproic acid, disulfiram
Thrombocytopenia (1). Drugs involved (4)	rosuvastatin, rivaroxaban, teicoplanin, tigecycline

Thrombotic microangiopathy (1). Drugs involved (3)	methylprednisolone, mycophenolic acid, tacrolimus
Disseminated intravascular coagulation (1). Drugs involved (2)	tigecycline, enoxaparin
**Skin and subcutaneous tissue disorders (29). Drugs involved (39) (31 different drugs)**	
Urticaria (6). Drugs involved (6)	methylprednisolone, acetylsalicylic acid, ciprofloxacin, cefazolin, nomegestrol and estradiol, erythromycin
Rash maculo-papular (4). Drugs involved (5)	cloxacillin, cefotaxime, iohexol, iomeprol, [illegible]
Rash pruritic (3). Drugs involved (5)	clindamycin, codeine combinations excl. psycholeptics, metamizole sodium, eslicarbazepine, [illegible]
Rash (3). Drugs involved (5)	ceftriaxone, allopurinol, clindamycin, daratumumab, methotrexate
Rash erythematous (2). Drugs involved (2)	cefazolin, pegaspargase
Erythema (2). Drugs involved (2)	ceftriaxone, rasburicase
Angioedema (2). Drugs involved (4)	paracetamol, ibuprofen, amoxicillin and beta-lactamase inhibitor, iohexol
Erythema multiforme (1). Drugs involved (1)	amoxicillin
Drug eruption (1). Drugs involved (3)	amoxicillin and beta-lactamase inhibitor, paracetamol, ibuprofen
Toxic skin eruption (1). Drugs involved (2)	trazodone, oxcarbazepine
Petechiae (1). Drugs involved (1)	meningococcus B, multicomponent vaccine
Stevens-Johnson syndrome (1). Drugs involved (1)	sulfamethoxazole and trimethoprim
Pruritus (1). Drugs involved (1)	metamizole sodium
Hyperhidrosis (1). Drugs involved (1)	immunoglobulins, normal human
**Respiratory, thoracic and mediastinal disorders (23). Drugs involved (33) (26 different drugs)**	
Respiratory failure (5). Drugs involved (9)	quetiapine (2), lorazepam, lithium, fluoxetine, diazoxide, methadone, clomethiazole, [illegible]
Acute interstitial pneumonitis (5). Drugs involved (5)	amiodarone (3), daptomycin, methotrexate
Pneumonitis (2). Drugs involved (3)	valproic acid, disulfiram, chloroquine
Apnea (2). Drugs involved (2)	pneumococcus, purified polysaccharides antigen; antilymphocyte immunoglobulin
Respiratory arrest (2). Drugs involved (4)	clonazepam, haloperidol, quetiapine, albumin
Alveolar proteinosis (1). Drugs involved (2)	sirolimus, tacrolimus
Hypersensitivity pneumonitis (1). Drugs involved (3)	sulfamethoxazole and trimethoprim, captopril, daptomycin
Respiratory depression (1). Drugs involved (1)	midazolam
Throat irritation (1). Drugs involved (1)	daratumumab
Hemoptysis (1). Drugs involved (1)	rivaroxaban
Laryngeal oedema (1). Drugs involved (1)	captopril
Cough (1). Drugs involved (1)	enalapril
**Vascular disorders (22). Drugs involved (39) (33 different drugs)**	
Abdominal wall hematoma (4). Drugs involved (9)	acenocoumarol (3), omeprazole, furosemide, enoxaparin, paroxetine, simvastatin, deflazacort
Pulmonary embolism (4). Drugs involved (5)	dienogest and ethinylestradiol, estradiol, etonorgestrel, olanzapine, progesterone
Hypotension (3). Drugs involved (3)	metamizole sodium, tizanidine, immunoglobulins, normal human

Hypertension (3). Drugs involved (5)	methylprednisolone, tacrolimus, mycophenolic acid, levonorgestrel, atorvastatin and ezetimibe
Cutaneous vasculitis (2). Drugs involved (4)	metamizole sodium, dexketoprofen, ceftriaxone, ampicillin
Hematoma muscle (2). Drugs involved (3)	acenocoumarol, atorvastatin, pantoprazole
Distributive shock (1). Drugs involved (6)	furosemide, enalapril, diazoxide, bisoprolol, tocilizumab, hydrochlorothiazide
Hemarthrosis (1). Drugs involved (2)	apixaban, sertraline
Re-opening of ductus arteriosus (1). Drugs involved (1)	diazoxide
Transverse sinus thrombosis (1). Drugs involved (1)	pegaspargase
**Renal and urinary disorders (19). Drugs involved (40) (20 different drugs)**	
Acute kidney injury (15). Drugs involved (33)	methotrexate (4), sulfamethoxazole and trimethoprim (2), ibuprofen (2), omeprazole (2), piperacillin and beta-lactamase inhibitor (2), ciclosporin (2), metformin, metamizole sodium, methylprednisolone, dexketoprofen, tacrolimus, furosemide, azithromycin, enalapril, paclitaxel, mycophenolic acid, losartan, cyclophosphamide, tizanidine, prednisone, spironolactone,
Renal tubular necrosis (1). Drugs involved (4)	ibuprofen, dexketoprofen, naproxen, diclofenac
Renal tubular disorder (1). Drugs involved (1)	ibuprofen
Immune-mediated nephritis (1). Drugs involved (1)	ibuprofen
Renal hemorrhage (1). Drugs involved (1)	acenocoumarol
**Immune system disorders (18). Drugs involved (22) (20 different drugs)**	
Anaphylactic shock (11). Drugs involved (13)	amoxicillin and beta-lactamase inhibitor (2), metamizole sodium, methylprednisolone, amiodarone, paclitaxel, midazolam, iohexol, levofloxacin, carboplatin, morphine, iodixanol,
Drug reaction with eosinophilia (5). Drugs involved (6)	sulfamethoxazole and trimethoprim, furosemide, lamotrigine, carbamazepine, allopurinol,
Hypogammaglobulinemia (1). Drugs involved (2)	carbamazepine, natalizumab
Cytokine release syndrome (1). Drugs involved (1)	iron, parenteral preparations
**Cardiac disorders (13). Drugs involved (25) (23 different drugs)**	
Atrioventricular block (4). Drugs involved (9)	sulfamethoxazole and trimethoprim, clonazepam, valproic acid, enalapril, propofol, ticagrelor, propranolol, flecainide, bisoprolol
Bradycardia (3). Drugs involved (5)	pneumococcus, purified polysaccharides antigen, midazolam, antilymphocyte immunoglobulin, lacosamide, fentanyl
Ventricular tachycardia (2). Drugs involved (6)	omeprazole, metformin, amiodarone, valsartan and sacubitril, eplerenone, tiotropium bromide
Tachycardia (1). Drugs involved (2)	propranolol, flecainide
Pericarditis (1). Drugs involved (1)	ulipristal
Myocarditis (1). Drugs involved (1)	prednisone
Prinzmental angina (1). Drugs involved (1)	capecitabine
**Investigations (5). Drugs involved (11) (8 different drugs)**	

International normalized ratio increased (1). Drugs involved (5)	amoxicillin and beta-lactamase inhibitor, acenocoumarol, methylprednisolone, mirtazapine, [illegible]
Coagulation test abnormal (1). Drugs involved (2)	tigecycline, enoxaparin
Blood fibrinogen decreased (1). Drugs involved (2)	tigecycline, enoxaparin
Electrocardiogram QT prolonged (1). Drugs involved (1)	amiodarone
Blood bilirubin increased (1). Drugs involved (1)	amoxicillin and beta-lactamase inhibitor
**Psychiatric disorders (8). Drugs involved (10) (6 different drugs)**	
Confusional state (4). Drugs involved (5)	ertapenem (2), quetiapine, tapentadol, cefepime
Suicide attempt (1). Drugs involved (1)	emtricitabine and tenofovir alafenamide and elvitegravir and cobicistat
Hallucinations (1). Drugs involved (2)	quetiapine, tapentadol
Disorientation (1). Drugs involved (1)	lorazepam
Irritability (1). Drugs involved (1)	lorazepam
**Infections and infestations (6). Drugs involved (11) (11 different drugs)**	
Pneumocystis jirovecii pneumonia (1). Drugs involved (3)	methotrexate, ciclosporin, prednisone
Disseminated tuberculosis (1). Drugs involved (2)	azathioprine, infliximab
Sepsis (1). Drugs involved (1)	pneumococcus, purified polysaccharides antigen
Balanitis candida (1). Drugs involved (1)	dapagliflozin
Meningitis listeria (1). Drugs involved (1)	alemtuzumab
Gastroenteritis rotavirus (1). Drugs involved (3)	methylprednisolone, vincristine, daunorubicin
**General disorders and administration site conditions (4). Drugs involved (6) (6 different drugs)**	
Oedema peripheral (2). Drugs involved (4)	tacrolimus, diazoxide, sirolimus, olmesartan
Generalized oedema (1). Drugs involved (1)	levonorgestrel
Pyrexia (1). Drugs involved (1)	meningococcus B, multicomponent vaccine
**Congenital, familial and genetic disorders (4). Drugs involved (8) (4 different drugs)**	
Neural tube defect (1). Drugs involved (3)	valproic acid, phenobarbital, levetiracetam
Hypoplastic left heart syndrome (1). Drugs involved (1)	lamotrigine
Arnold-Chiari malformation (1). Drugs involved (2)	valproic acid, lamotrigine
Meningomyelocele (1). Drugs involved (2)	valproic acid, lamotrigine
**Musculoskeletal and connective tissue disorders (4). Drugs involved (4) (2 different drugs)**	
Immune-mediated myositis (3). Drugs involved (3)	atorvastatin (3)
Muscular weakness (1). Drugs involved (1)	zoledronic acid
**Eye disorders (2). Drugs involved (2) (2 different drugs)**	
Photopsia (1). Drugs involved (1)	ivabradine
Diplopia (1). Drugs involved (1)	topiramate
**Injury, poisoning and procedural complications (1). Drugs involved (1)**	
Infusion related reaction (1). Drugs involved (1)	amiodarone
**Neoplasms benign, malignant and unspecified (incl. cyst and polyps) (1). Drugs involved (3) (3 different drugs)**	
Leukemia (1). Drugs involved (3)	paclitaxel, carboplatin, radiotherapy
**Pregnancy, puerperium and perinatal conditions (1). Drugs involved (5) (5 different drugs)**	
Oligohydramnios (1). Drugs involved (5)	cyclophosphamide, doxorubicin, paclitaxel, pertuzumab, trastuzumab

Table S2. Most frequently reported adverse drug reactions by organ class system.

System organ class (SOC) disorders	Total ADR
Nervous system disorders	63 (17.4)
Metabolism and nutrition disorders	38 (10.5)
Gastrointestinal disorders	36 (9.9)
Hepatobiliary disorders	35 (9.6)
Blood and lymphatic system disorders	31 (8.5)
Skin and subcutaneous tissue disorders	29 (8.0)
Respiratory, thoracic and mediastinal disorders	23 (6.3)
Vascular disorders disorders	22 (6.1)
Renal and urinary disorders	19 (5.2)
Immune system disorders	18 (5.0)
Cardiac disorders	13 (3.6)
Psychiatric disorders	8 (2.2)
Infections and infestations	6 (1.7)
Investigations	5 (1.4)
General disorders and administration site conditions	4 (1.1)
Congenital, familial and genetic disorders	4 (1.1)
Musculoskeletal and connective tissue disorders	4 (1.1)
Eye disorders	2 (0.6)
Injury, poisoning and procedural complications	1 (0.3)
Pregnancy, puerperium and perinatal conditions	1 (0.3)
Neoplasms benign, malignant and unspecified (incl. cysts and polyps)	1 (0.3)
Total	363 (100)

*More than one adverse drug reaction could be notified in each report of suspected adverse drug reactions.

Table S3. Most frequently adverse drug reactions.

Adverse drug reaction (Preferred Term)	n	%
Hepatic cytolysis	17	4.7
Acute kidney injury	15	4.1
Anaphylactic shock	11	3
Lactic acidosis	11	3
Vomiting	9	2.5
Somnolence	8	2.2
Hepatitis fulminant	7	1.9
Depressed level of consciousness	6	1.7
Hepatitis cholestatic	6	1.7
Hyperkalaemia	6	1.7
Metabolic acidosis	6	1.7
Pancytopenia	6	1.7
Urticaria	6	1.7
Acute interstitial pneumonitis	5	1.4
Agranulocytosis	5	1.4
Diarrhoea	5	1.4
Drug reaction with eosinophilia and systemic	5	1.4
Respiratory failure	5	1.4
Others	224	61.7
Total	363	100

*More than one adverse drug reaction could be notified in each report of suspected adverse drug reactions.

Table S4. Most frequently drugs involved in the adverse drug reactions by ATC subgroup.

Therapeutic subgroup	n	%
Antibacterials for systemic use (J01)	70	14.4
Psycholeptics (N05)	46	9.4
Antineoplastic agents (L01)	44	9.0
Immunosuppressants (L04)	38	7.9
Antiepileptics (N03)	31	6.4
Antithrombotic agents (B01)	30	6.2
Analgesics (N02)	26	5.4
Antidepressants (N06)	24	4.8
Antiinflammatory and antirheumatic products (M01)	17	3.5
Drugs used in diabetes (A10)	15	3.1
Drugs for acid related disorders (A02)	14	2.9
Agents acting on the renin-angiotensin system (C09)	13	2.7
Corticosteroids for systemic use (H02)	13	2.7
Lipid modifying agents (C10)	12	2.5
Cardiac therapy (C01)	10	2.1
Diuretics (C03)	9	1.9
Sex hormones and modulators of the genital system (G03)	9	1.9
Antimycobacterials (J04)	6	1.2
Contrast media (V08)	6	1.2
Beta blocking agents (C07)	5	1.0
All other therapeutic products (V03)	5	1.0
Others	44	8.8
Total	487*	100

*A total of 207 different drugs were involved in the adverse drugs reactions but a drug could be involved in more than one adverse drug reaction.

Table S5. Most frequently drugs involved in adverse drug reactions.

Drug	n	%
quetiapine	17	3.5
sulfamethoxazole and trimethoprim	13	2.7
acenocoumarol	12	2.5
metamizole sodium	11	2.3
metformin	11	2.3
methotrexate	11	2.3
omeprazole	11	2.3
amoxicillin and beta-lactamase inhibitor	10	2.1
paracetamol	10	2.1
amiodarone	8	1.6
atorvastatin	8	1.6
methylprednisolone	8	1.6
tacrolimus	8	1.6
ibuprofen	7	1.4
pegaspargasa	7	1.4
dexketoprofen	6	1.2
ertapenem	6	1.2
furosemide	6	1.2
mirtazapine	6	1.2
valproic acid	5	1
lorazepam	5	1
Others	301	61.8
Total	487*	100

*A total of 207 different drugs were involved in the adverse drugs reactions but a drug could be involved in more than one adverse drug reaction.
